# Encapsulation and Immobilization of Functional Molecules Using Cage-Like Porous Frameworks

**DOI:** 10.1021/acsami.6c02300

**Published:** 2026-05-05

**Authors:** Hiroi Sei, Yukako Fujita, Yuta Tanaka, Hitoshi Kasai, Kouki Oka

**Affiliations:** † Institute of Multidisciplinary Research for Advanced Materials, 13101 Tohoku University, 2-1-1 Katahira, Aoba-ku, Sendai, Miyagi 980-8577, Japan; ‡ Center for the Promotion of Interdisciplinary Education and Research, Kyoto University, Yoshida-honmachi, Sakyo-ku, Kyoto 606-8501, Japan; § Carbon Recycling Energy Research Center, Ibaraki University, 4-12-1 Nakanarusawacho, Hitachi, Ibaraki 316-0033, Japan

**Keywords:** immobilization, encapsulation, porous frameworks, luminescent, catalyst, drug

## Abstract

To exhibit the intrinsic functions of functional molecules not only in solution but also in solid materials, we have developed immobilization methods that use porous frameworks as support materials. Conventionally, functional molecules are immobilized via impregnation into porous frameworks using physisorption or grafting on the pore surfaces using chemical bonds. However, these methods can lead to leakage during use and performance degradation owing to structural modifications for immobilization. Recently, cage-like porous frameworks have attracted attention as support materials, and a novel method (encapsulation), where the functional molecules are reliably confined into the pores, has been discovered. The narrow windows of cage-like porous frameworks physically confine functional molecules into their pores, thereby suppressing leakage without chemical bonds and preserving the intrinsic function of the molecules after immobilization. In this perspective, we comprehensively summarize various encapsulation methods and developments of functional solid materials based on encapsulation and outline their future prospects.

## Graphical abstract

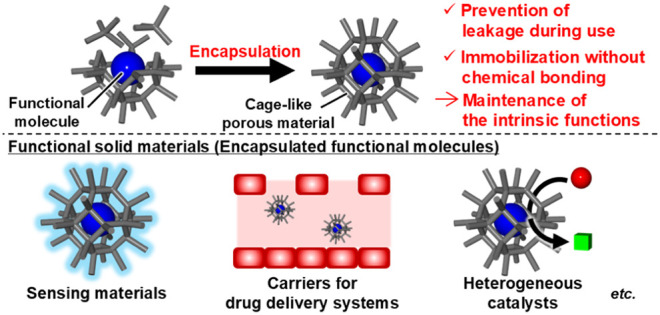

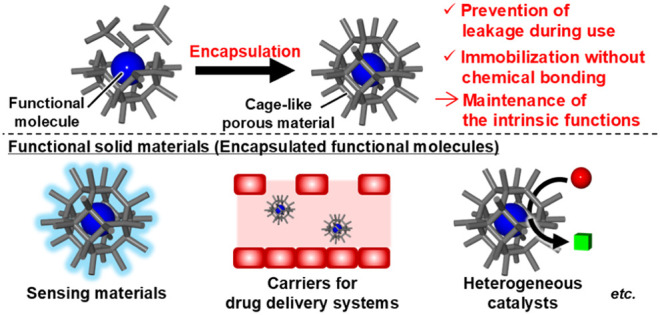

## 1 Introduction

For most functional molecules that exhibit their functions in solutions,^1–3^ their aggregation causes loss of function or performance degradation.^4–6^ However, in practical applications, these molecules are often used as solid materials, such as powders,^7^ thin films,^8^ and nanoparticles.^9^ To enable the exhibition of their intrinsic functions in solid materials, functional molecules are dispersed and immobilized in support materials. The immobilization of functional molecules such as luminescent dyes, drugs, and molecular catalysts has led to the development of functional solid materials—sensing materials,^10^ carriers for drug delivery systems (**DDS**),^11^ and heterogeneous catalysts^12^—in various research fields.

Highly periodic porous frameworks, such as mesoporous silica,^13,14^ metal–organic frameworks (**MOF**s),^10,15^ covalent organic frameworks (**COF**s),^16^ and hydrogen-bonded organic frameworks (**HOF**s),^17,18^ have high surface areas, controllable pore sizes, and designability of pore surfaces; therefore, they have attracted significant attention as support materials for the immobilization of functional molecules. As a representative immobilization method with porous frameworks, the impregnation method, shown in Figure 1 left, is widely known.^14,19,20^ The immersion of porous frameworks with relatively large pores in a solution of functional molecules allows the molecules to be physisorbed into the pores, which enables facile immobilization of functional molecules. In addition, as shown in Figure 1 center, the grafting method, where the functional molecule is incorporated into the structure of the porous framework by chemical bonding, is also well-known.^13,21,22^

**Figure 1:**
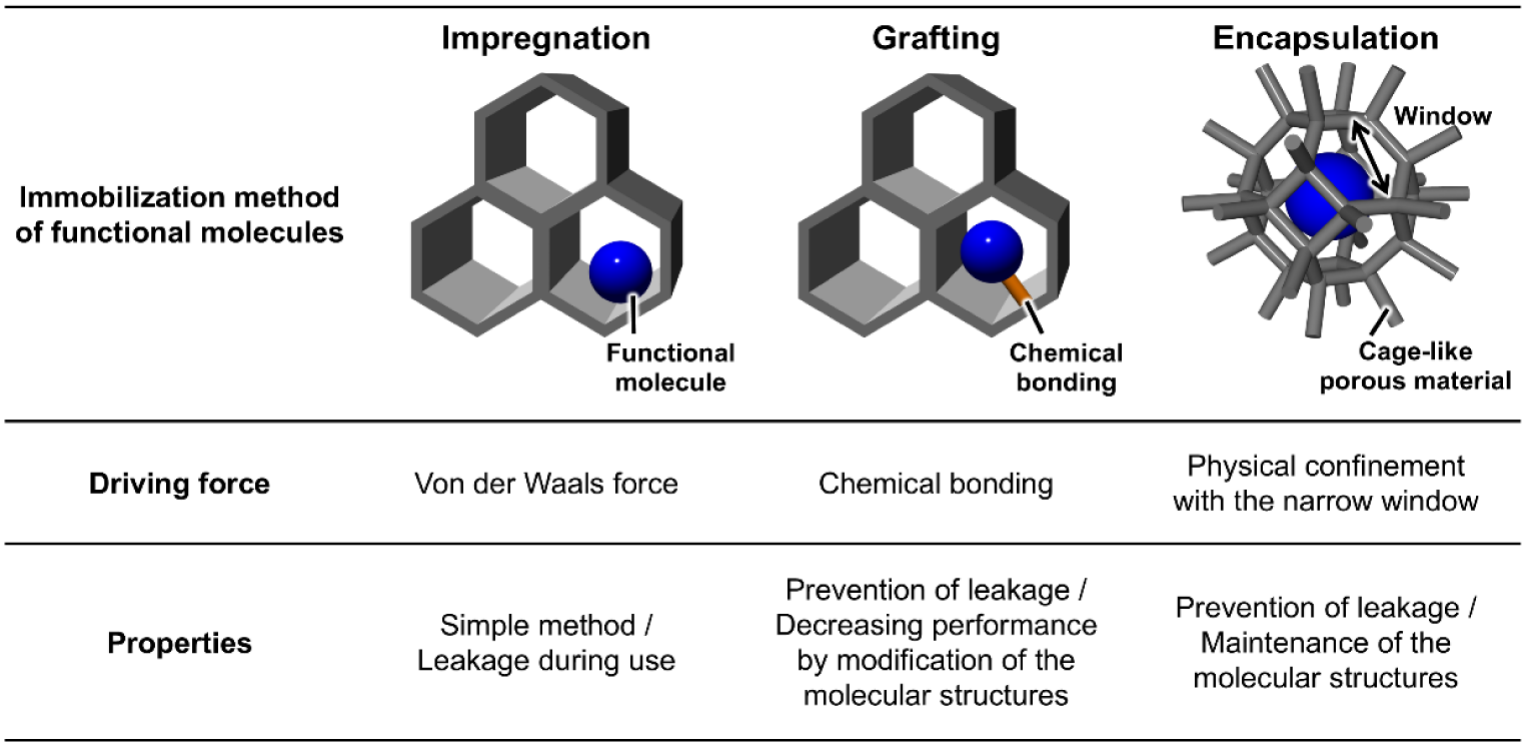
Comparison of the immobilization methods of functional molecules.

However, in the former method, functional molecules are immobilized into the support materials only by van der Waals forces, and therefore, the molecules often leak during use.^12,20,23^ In the latter method, for the formation of a chemical bond between the porous framework and functional molecule, modification of the molecule is necessary, which leads to performance degradation.^11,12,24^

To solve these challenges, a novel immobilization method, where cage-like porous frameworks are used as supporting materials and functional molecules are reliably confined into the pores, has been discovered (Figure 1 right).^25,26^ Cage-like porous frameworks have larger inner pores and narrower windows (Figure 1 right) than the sizes of functional molecules. The narrow window confines the molecules into the inner pores (encapsulation), which suppresses leakage without chemical bonding. In addition, by the suppression of leakage, the cage-like porous frameworks can retain the functions of immobilized molecules for a longer period than typical 3D porous frameworks (Table S1).

Previous reviews have focused on immobilization methods that can be applied to porous frameworks of any structure. In this perspective, for the first time, we focus on the encapsulation of functional molecules within cage-like porous frameworks, independent of chemical bonding or chemical interactions between the molecule and the framework. Section 2 outlines the encapsulation strategies and their characteristics. Section 3 introduces recent advances in the encapsulation of functional molecules such as luminescent dyes, drugs, and molecular catalysts, which are commonly targeted for immobilization, and the development of functional solid materials based on encapsulation. Section 4 summarizes the progress and challenges and outlines the directions for future research.

## 2 Encapsulation Strategies

As shown in Figure 2, functional molecules are encapsulated into cage-like **MOF**s and **HOF**s using various strategies (Table 1). Cage-like **COF**s have not yet been applied to the encapsulation of luminescent dyes, drugs, and molecular catalysts. This is due to the large window sizes of ambient-stable examples relative to many functional molecules and their rarity, arising from the difficulty of structural determination.^27^ This section outlines the characteristics of emerging encapsulation strategies: the bottle-around-ship strategy^28,29^ (Figure 2a) in Section 2.1, the ship-in-a-bottle strategy^28,29^ (Figure 2b) in Section 2.2, and the aperture-opening strategy^30^ (Figure 2c) in Section 2.3. In Section 2.4, an up-to-date pressure-driven strategy is introduced (Figure 2d).^25^ In addition, the requirements for each functional molecule (luminescent dye, drug, and molecular catalyst) as well as suitable encapsulation strategies are summarized in Table 2. For luminescent dyes, increasing loading ratio causes a decrease in the quantum yield due to the quenching with more than one dye molecule in a pore. Loading ratios up to 40% exhibit higher quantum yields compared to those of higher loading ratios.^31^ Therefore, for luminescent dyes, a middle loading ratio is desirable. For drugs to achieve superior therapeutic efficacies, a high loading ratio is desirable. For molecular catalysts, to avoid diffusion inhibition of reactants and products, a low loading ratio is desirable. In the following section, we outline which encapsulation strategy is most suitable for each molecule.

**Table 1:** Main Cage-like Porous Frameworks Used for Encapsulation

Cage-like porous frameworks		Window/Inner pore (Å)	Encapsulation strategies	Ref
**MOF**s	**ZIF-8**	3.4/12	Bottom-around-ship, Ship-in-a-bottle, Aperture-opening	^30,32,33^
	**ZIF-20**	2.8/15	Bottom-around-ship	^34^
	**ZIF-71**	4.2/17	Bottom-around-ship	^33^
	**ZIF-90**	3.5/11	Bottom-around-ship	^35^
	**UiO-66**	5.3/11	Bottom-around-ship, Ship-in-a-bottle, Aperture-opening	^36–38^
	**UiO-67**	8.1/16	Ship-in-a-bottle, Aperture-opening	^38,39^
	**MIL-100**	5.0/24, 8.6/29	Ship-in-a-bottle	^40^
**HOF**s	**q-TO**	7.0/12	Bottom-around-ship	^41^
	***s*** **-POS**	5.2/21	Bottom-around-ship	^42–44^

**Figure 2:**
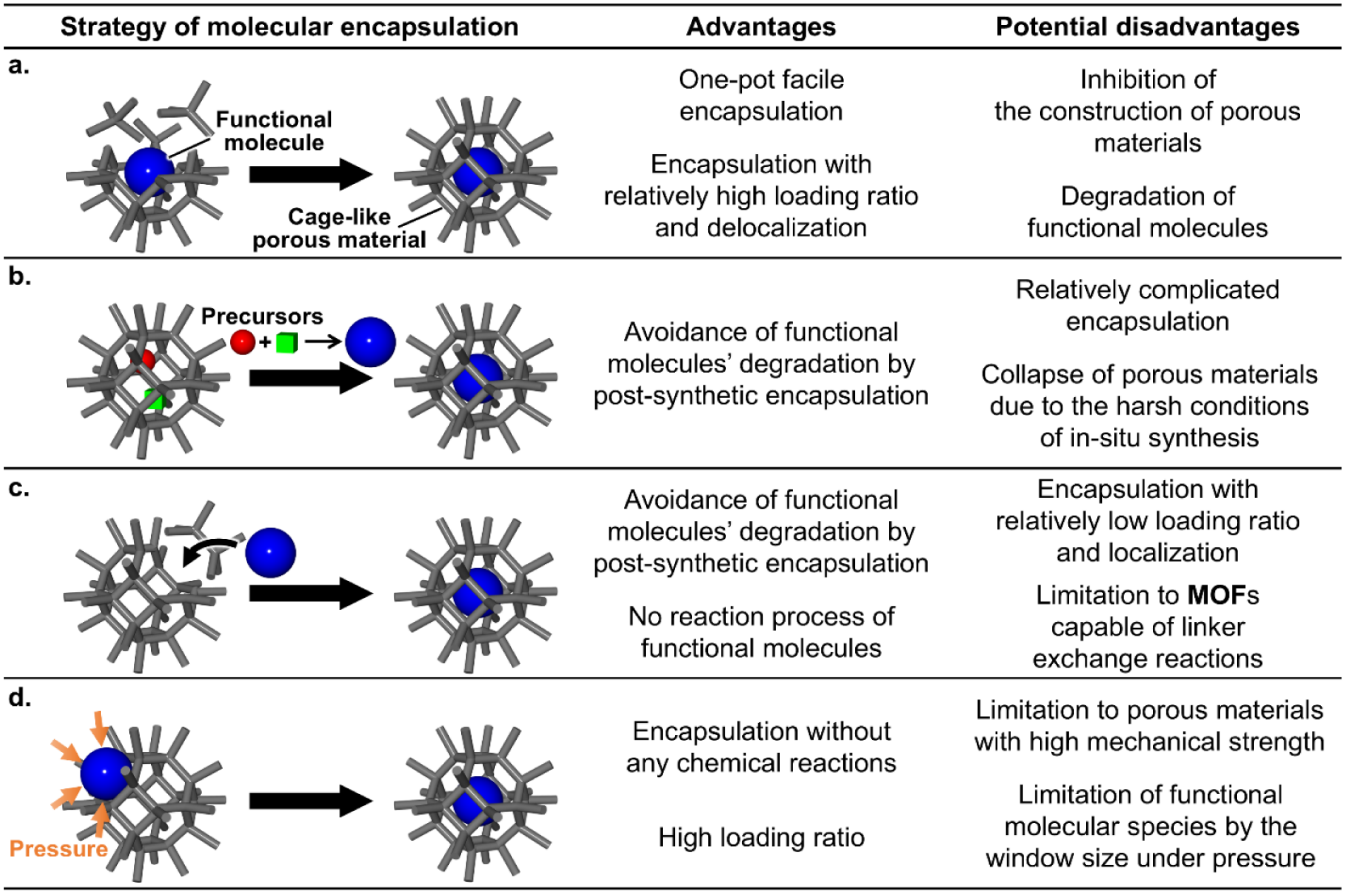
Schematic representations and comparison of molecular encapsulation strategies: (a) bottle-around-ship strategy, (b) ship-in-a-bottle strategy, (c) aperture-opening strategy, and (d) pressure-driven strategy.

**Table 2:** Encapsulation of Luminescent Dyes, Drugs, and Molecular Catalysts: Requirements for Porous Frameworks, a Proper Loading Ratio, and Suitable Strategies

Functional molecules	Requirements for porous frameworks	Proper loading ratio^a^	Suitable encapsulation strategy
Luminescent dye	Nonluminescent	Middle (1–40%)^31^	Bottle-around-ship
Drug	Biocompatible, low toxic	High (>100%)	Bottle-around-ship, Pressure-driven
Molecular catalyst	Chemical stable	Low (0.1–1%)^45^	Ship-in-a-bottle, Aperture-opening

a The number of functional molecules per a cage of the porous framework.

### 2.1 Bottle-Around-Ship Strategy

As shown in Figure 2a, in the bottle-around-ship strategy, the addition of functional molecules to the solvent during the synthesis of cage-like porous frameworks enables the construction of a porous structure around the molecules and their encapsulation. This strategy enables the encapsulation of functional molecules via a one-pot process, making it the most facile strategy. In addition, this strategy uniformly immobilizes the molecules in the porous framework and exhibits a relatively high loading ratio. However, it has potential disadvantages. Functional molecules may inhibit the synthesis of cage-like porous frameworks, and exposure to the synthesis conditions of porous frameworks can result in degradation or decomposition of the functional molecules.

Most **MOF**s are synthesized under high-pressure and high-temperature conditions.^46^ Ambient condition synthesis of **MOF**s by mixing solutions of metal ions and linkers at ambient conditions has been developed. In particular, divalent **MOF**s tend to be easily synthesized at ambient condition owing to the moderate coordination bond strength,^46^ and the development has focused on zeolitic imidazolate frameworks (**ZIF**s),^47^ which are cage-like **MOF**s composed of divalent metal ions and imidazole derivatives.^48–50^ Therefore, in **ZIF**s, ambient condition encapsulation of functional molecules via the bottle-around-ship strategy has been developed.^51,52^ **HOF**s are organic porous frameworks with hydrogen bonding that are synthesized under mild conditions such as recrystallization.^53,54^ Therefore, in cage-like **HOF**s, the adaptation of the encapsulation of various functional molecules using the bottle-around-ship strategy is expected, and the encapsulation of luminescent dyes—functional molecules that are easy to evaluate—into cage-like **HOF**s was reported.^42–44^

Luminescent dyes have rigid π-conjugated skeletons and high thermal stabilities, which make them suitable functional molecules for this encapsulation strategy. This strategy achieves a relatively high loading ratio, which is advantageous for drug encapsulation. In contrast, some metal complex catalysts—a major class of molecular catalysts—decompose at the synthesis temperatures of **MOF**s and form metal precipitates,^29^ which limits the applicability of this strategy to the molecular catalysts.

### 2.2 Ship-In-a-Bottle Strategy

As shown in Figure 2b, in the ship-in-a-bottle strategy, functional molecules are synthesized *in situ* in inner pores and encapsulated into cage-like porous frameworks. Therefore, the precursors of these molecules must be introduced in the pores using the impregnation method or bottle-around-ship strategy in advance. This strategy involves postsynthetic encapsulation, in which functional molecules are confined after the synthesis of cage-like porous frameworks; therefore, the degradation or decomposition of the molecules is avoidable. However, the ship-in-a-bottle strategy requires the introduction of precursors into pores, which complicates the encapsulation process. In addition, when introducing a precursor using a bottle-around-ship strategy, high thermal stability is required. Under harsh *in situ* synthesis conditions, the collapse of the porous structure may be a potential disadvantage of this strategy.

Several **MOF**s exhibit chemical stability, making them suitable for the ship-in-a-bottle strategy. While chemically stable **MOF**s, such as **UiO-66**, maintain their structures even in organic solvents and under acidic conditions, **HOF**s, which are constructed with relatively weak hydrogen bonds, usually become structurally unstable in polar solvents and acidic conditions. Therefore, the porous structure may collapse using this strategy. Consequently, encapsulation into cage-like **HOF**s has been limited to the bottle-around-ship strategy. Molecular catalysts can be synthesized through relatively simple reactions, such as acid–base neutralization and complex formation, which makes them suitable for this strategy. Many luminescent dyes and drugs have complicated molecular structures that make *in situ* synthesis difficult; therefore, this strategy is unsuitable for their molecules.

### 2.3 Aperture-Opening Strategy

As shown in Figure 2c, the aperture-opening strategy encapsulates functional molecules via a temporary window expansion that is caused by the exchange reaction of dissociative linkers in cage-like **MOF**s. As shown in Figure 3, this strategy involves the following processes: (1) temporary window expansion via linker dissociation, (2) introduction of functional molecules into the inner pores of the cage-like **MOF**s through the expanded window, and (3) restoration of the window size by linker association and encapsulation of the molecules.

**Figure 3:**
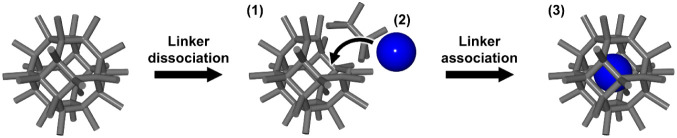
Schematic representation of processes in the aperture-opening strategy: (1) Temporary window expansion, (2) introduction of functional molecules, and (3) restoration of the window size and encapsulation of the molecules.

The aperture-opening strategy was demonstrated by Tsung et al. in 2014 ^30^ and has been applied to the encapsulation of luminescent dyes and metal complex catalysts.^55,56^ This strategy involves postsynthetic encapsulation, which enables the encapsulation of functional molecules without their degradation or decomposition. In addition, unlike in the ship-in-a-bottle strategy, functional molecules are encapsulated without reaction processes; therefore, the potential collapse of the porous structure is avoidable. In contrast, encapsulation proceeds from the surface of the porous framework crystal, and therefore, immobilized functional molecules localize near the surface.^26^ In addition, because the molecules are immobilized into pores near the crystal surface, the loading ratio is relatively low (<2 wt %).^56^

This strategy requires a linker exchange reaction, which limits its application to **MOF**s. However, some **MOF**s cannot undergo linker exchange reactions,^57^ and this strategy has only been applied to certain cage-like **MOF**s such as **UiO-66**, **UiO-67**, and **ZIF-8** (Table 1).

In molecular catalysts, this strategy enables encapsulation without degradation, which makes it suitable. For metal complex catalysts with complicated coordination environments, because the catalysts are encapsulated with maintenance of their structures, this strategy is considered superior to the ship-in-a-bottle strategy. However, in the case of luminescent dyes and drugs, a low loading ratio may lead to a decrease in luminescent intensities and a low drug-loading capacity; therefore, this strategy is unsuitable for their encapsulation.

### 2.4 Pressure-Driven Strategy

The pressure-driven strategy is a novel encapsulation strategy reported by Tao Li et al. in 2025.^25^ As shown in Figure 2d, this strategy utilizes localized structural changes in cage-like porous frameworks under pressure to encapsulate liquid-state functional molecules. As shown in Figure 4, this strategy involves the following processes: (1) expansion of porous structures and an increase in the window size of cage-like porous frameworks through linker rearrangement under pressure, (2) diffusion of functional molecules into inner pores through the window, and (3) restoration of the window size upon return to atmospheric pressure and encapsulation of functional molecules.

**Figure 4:**
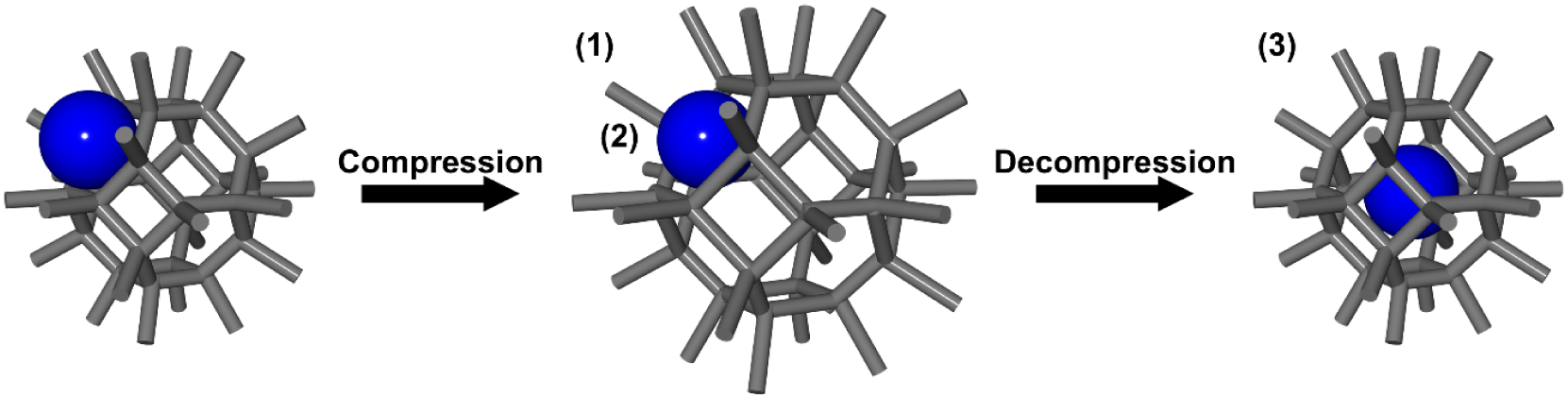
Schematic representation of processes in the pressure-driven strategy: (1) expansion of porous structures and an increase in the window size, (2) diffusion of functional molecules, and (3) restoration of the window size and encapsulation of the molecules.

Li et al. used **UiO-66** and 1-Hexyl-3-methylimidazolium bromide (**HmimBr**), which is a molecular catalyst for CO_2_ fixation, and demonstrated this strategy.^25^ By applying a pressure of 9.5 MPa to a mixture of these materials, **HmimBr** was encapsulated into **UiO-66** and a high loading ratio of 59 wt %, which is 91% of the theoretical maximum loading ratio (64.6 wt %), was achieved. This strategy encapsulates functional molecules without chemical reaction processes of preparation of cage-like porous frameworks. In addition, this strategy requires no additional solvent for encapsulation, and the application of pressure facilitates the diffusion of functional molecules into the porous framework, which enables encapsulation with a high loading ratio. In contrast, because this strategy requires the application of pressure to porous frameworks, mechanically robust materials such as **MOF**s are preferable. In addition, the functional molecules that can be encapsulated are limited not only by the size of the inner pores but also by the maximum size of the window under pressure. Moreover, the maximum size is determined by high-pressure crystallography,^58^ and only a few cage-like porous frameworks, such as **ZIF-8,** have been investigated.

This strategy enables the encapsulation of functional molecules without the chemical reaction processes of preparation of cage-like porous frameworks and is expected to be applied to the encapsulation of various functional molecules. Because encapsulation with a high loading ratio is possible, this strategy is considered to be particularly useful for drug encapsulation.

### 2.5 Summary of Section 2

The bottle-around-ship strategy is the primary encapsulation strategy. However, to avoid the degradation of functional molecules during encapsulation, the use of cage-like porous frameworks synthesized under mild conditions and the development of postsynthetic encapsulation strategies have advanced. **MOF**s have been utilized in numerous studies as support materials for the encapsulation of functional molecules, but the **MOF**s that can be synthesized under ambient conditions are limited. In addition, among the **HOF**s that can be synthesized under relatively mild conditions, cage-like porous structures suitable for encapsulation are rare, and their design guidelines have not been established.

The ship-in-a-bottle, aperture-opening, and pressure-driven strategies have been developed as postsynthetic encapsulation strategies. These strategies exhibit different loading ratios and distributions of functional molecules in the crystal, which are considered to affect their performance. Therefore, comparative experiments on the performance between these strategies are highly desired to achieve the exhibition of the intrinsic function of functional molecules.

## 3 Recent Investigations into the Encapsulation of Functional Molecules

This section focuses on functional molecules, such as luminescent dyes, drugs, and molecular catalysts, which are commonly targeted for immobilization. Sections 3.1, 3.2, and 3.3 outline the recent progress in functional solid materials based on the encapsulation of luminescent dyes, drugs, and molecular catalysts, respectively.

### 3.1 Encapsulation of Luminescent Dyes

Organic luminescent dyes exhibit fluorescence upon light absorption and are widely used as solid-state luminescent materials, such as sensing materials and light emitting layer of organic light-emitting diodes.^59^ By molecular design, these dyes enable the control of the luminescent wavelength and selective luminescence toward specific targets. However, this luminescence is quenched in high-concentration solvents and in the aggregated solid state; therefore, the quantum yield is significantly reduced.^8^ For overcoming this challenge, the dyes have been dispersed and immobilized in support materials to suppress the aggregation, and solid-state luminescent materials have been developed.^10,60^

Encapsulation of dyes has been considered to suppress their non-radiative deactivation and enhance their luminescent properties, such as fluorescence lifetime.^61^ Recently, the effect of encapsulation on the fluorescence lifetimes was investigated in detail. As shown in Figure 5, Hongyu Chen et al. encapsulated various luminescent dyes (Figure 5a) into **ZIF-8** (Figure 5b) and **ZIF-71** (Figure 5b) to investigate the relationship between dye’s size, conformation, and their fluorescence lifetimes.^33^ As shown in Figure 5d, **Dye 9** encapsulated in **ZIF-8** and **ZIF-71** exhibited fluorescence lifetimes longer than that of the free-state dye. However, in the case of **Dye 1** (12 Å × 13 Å) shown in Figure 5c, **Dye 1** encapsulated into **ZIF-71** with large inner pores exhibited a fluorescence lifetime comparable to that of the free-state dye, but **Dye 1** encapsulated into **ZIF-8** exhibited a shorter lifetime than that of the free-state dye. As shown in Figure 5e, the molecular dynamics simulation indicated that the chromophore of **Dye 1** encapsulated into **ZIF-8** underwent buckling. The authors reported for the first time that when the sizes of the dyes are larger than those of the inner pores, their fluorescence lifetimes increase if the substituents of the chromophore undergo folding but decrease if the chromophore undergoes buckling.

**Figure 5:**
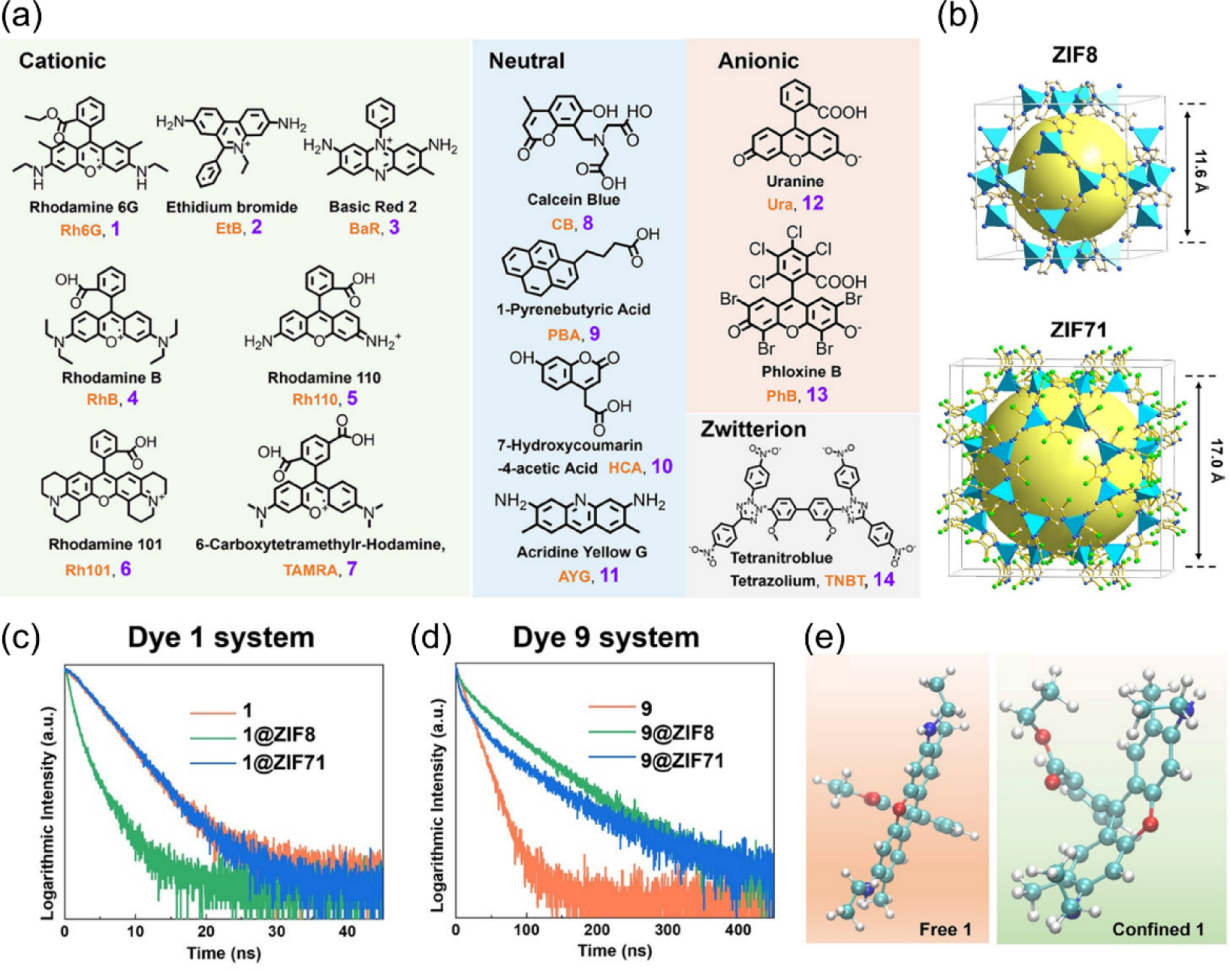
(a) 14 organic dyes. (b) Schematic representation of the unit cell of **ZIF-8** and **ZIF-71**. Transient fluorescence decay traces of (c) **Dye 1** and (d) **Dye 9** in different confined environments: free state (orange trace), confined within **ZIF-8** (green trace), and confined within **ZIF-71** (blue trace). (e) Representative conformations in the two states are shown for the free and confined states. Reproduced from ref. 33. Available under a CC-BY license. Copyright 2025, X. Xiao, Q. Hong, X. Yan, R. Liu, Y. Wu, C. Li, B. Gu, G. He, and H. Chen, published by Wiley-VCH.

In addition, changes in the luminescence phenomenon of dyes due to encapsulation were reported. As shown in Figure 6a, we used tetrasulfonic acid with bulky adamantane core (**AdPS**) and **TPMA-X**, where heavy atoms (halogen) were introduced in the *para*-position of benzene rings of **TPMA**, to construct cage-like **HOF**s (***s*** **-POS**s) with heavy-atom effects, and the luminescent dyes such as pyrene and coronene were encapsulated using the bottle-around-ship strategy.^42,44^ As shown in Figure 6b and c, the external heavy-atom effect that promotes intersystem crossing and the adsorption property that does not adsorb oxygen, a quencher, in ***s*** **-POS**s successfully induced the room-temperature phosphorescence of encapsulated coronene (12 Å × 12 Å) in air.^43,44^

**Figure 6:**
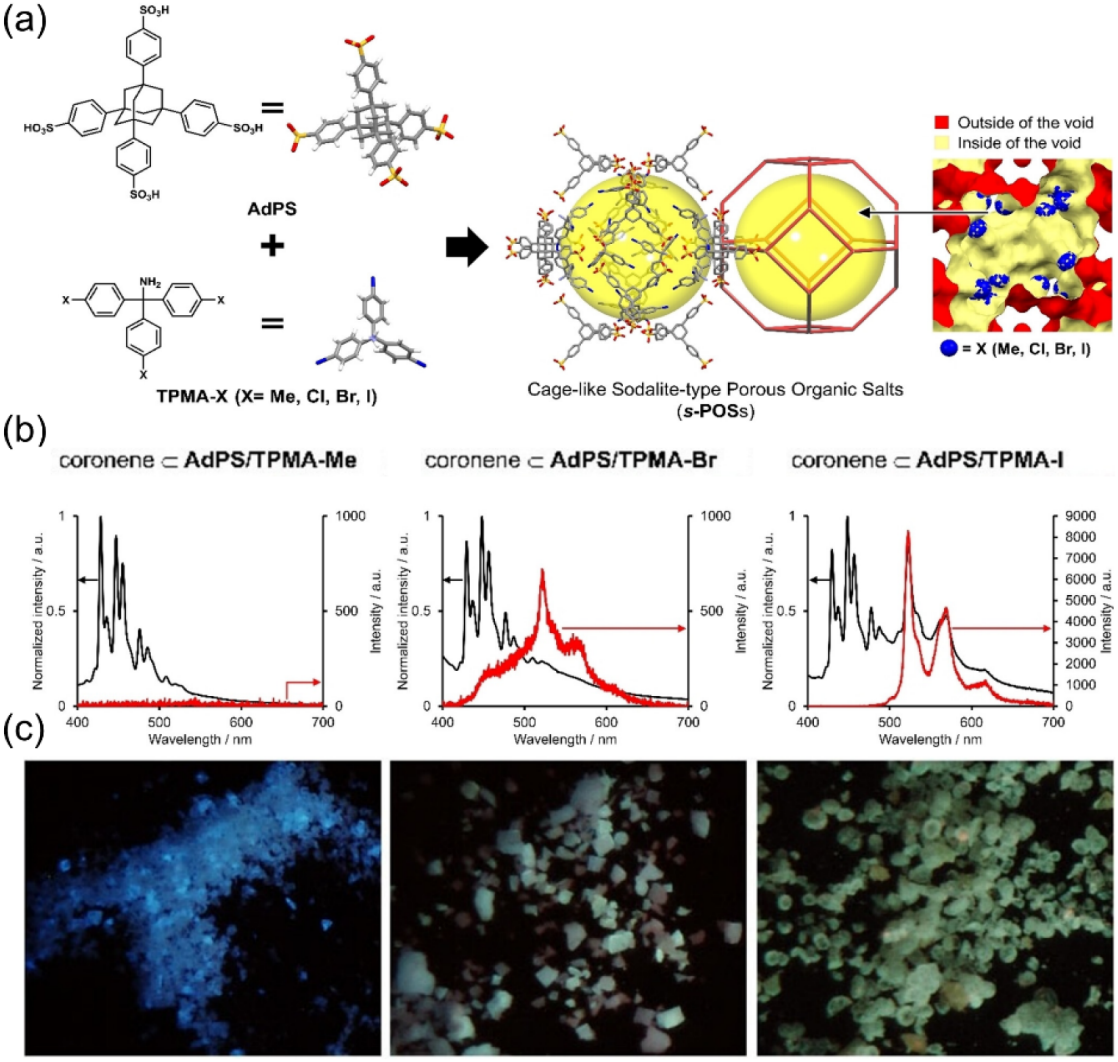
(a) ***s*** **-POS**s constructed from **AdPS** and **TPMA-X** (**X = Me, Cl, Br, or I**). (b) Steady-state photoluminescence (black lines) and phosphorescence (red lines) spectra (excitation at 340 nm). (c) Photographs (under UV irradiation (λ = 340 nm)) of the encapsulated coronene. Reproduced with permission from ref. 44. Copyright 2023 Wiley-VCH.

To date, various luminescent dyes have been encapsulated to exhibit their luminescence properties in the solid state. In these systems, the effect of encapsulation on the luminescence properties has been discovered. This mechanism is considered to involve the conformation of the dye, and therefore, elucidation of the specific conformation of the encapsulated dye by advanced measurements, such as X-ray crystallography, pair distribution function analysis, and electron diffraction structure analysis, is desired. In addition, changes in the luminescence phenomenon of encapsulated dyes due to the pore environment and the adsorption properties of porous frameworks were reported.^42,44^ Although multiple factors are likely to influence the luminescence phenomenon, detailed elucidation of the mechanisms through dynamic measurements, such as time-resolved transient absorption spectroscopy, has not been achieved.

### 3.2 Encapsulation of Drugs

Drugs exhibit therapeutic activity and are essential for treating various diseases, such as tumors and infections. However, because of the low target specificity of free-state drugs, the majority fail to reach the target tissue, which results in a reduction in therapeutic efficacy.^11,62^ In addition, the delivery of drugs to healthy cells leads to an increase in side effects.^11,62^ To enhance therapeutic efficacy and reduce side effects, drug delivery systems (**DDS**) using nanocarriers with drugs have been proposed.^63^ Passive targeting based on particle size or active targeting based on surface modification of the carrier enhances targeting specificity, which enables drug release in the target tissue. To realize this system, drugs have been immobilized in support materials, and improvements in drug-loading capacity and the development of nanocarriers that exhibit stimulus-responsive drug release have been investigated.^11^

To date, several drugs have been immobilized in single **MOF**s. As shown in Figure 7a, Amur et al. encapsulated Glabridin (**Glab**), a representative antibacterial drug, into **ZIF-8** to prepare **Glab@ZIF-8** using the bottle-around-ship strategy for preparation of nanocarriers with the antibacterial activity of **Glab**.^64^ In a drug-release assay at pH = 5.0, the release of Glab at 25 °C was only 3% over 6 h, indicating that Glab was encapsulated within the inner pores of **ZIF-8**. As shown in Figure 7b, in antibacterial study, **Glab@ZIF-8** exhibited higher antibacterial activity than that of either **ZIF-8** or **Glab** due to their synergistic antibacterial effects that contain damages of the bacterial cell wall by **ZIF-8** and the antibacterial action of **Glab**. In addition, **Glab@ZIF-8** exhibits antibacterial activity comparable to that of the standard antibiotic ampicillin (Figure 7b).

**Figure 7:**
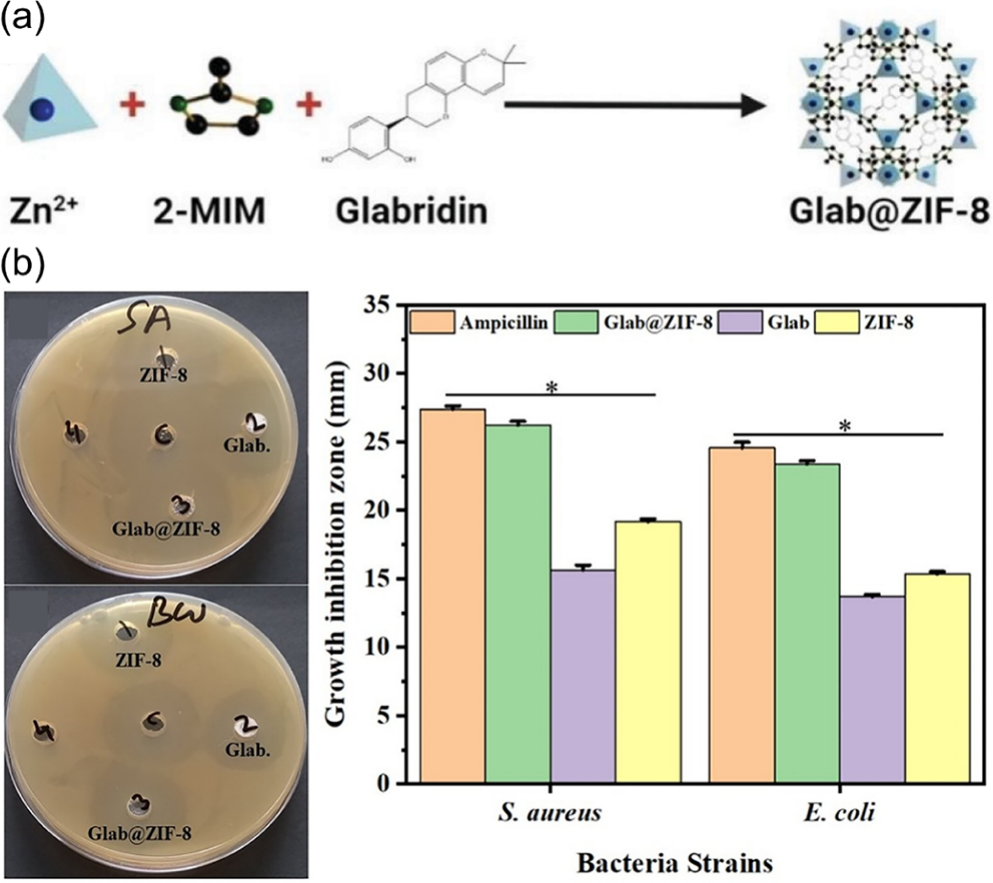
(a) Schematic representation of the preparation of **Glab@ZIF-8**. (b) Antibacterial activities of **Glab@ZIF-8**, **ZIF-8**, **Glab**, and ampicillin against *S. aureus* and *E. coli*. Reproduced with permission from ref. 64. Copyright 2025 Wiley-VCH.

Nanocarriers that can be controlled to release encapsulated drugs in response to multiple stimuli have been developed. Ribeiro et al. constructed a core–shell nanocomposite (**UCNP@ZIF-8-DOX**, Figure 8) by forming a shell of **ZIF-8**, where the anticancer drug doxorubicin (**DOX**, 6.9 Å × 15 Å) was encapsulated using the bottle-around-ship strategy around the core of a lanthanide-doped upconversion nanoparticle (**UCNP**) that can convert two low-energy photons in the near-infrared region into one high-energy photon.^65^ A drug release assay at pH = 7.4 demonstrated that **DOX** was encapsulated into the inner pores of **ZIF-8**. **UCNP@ZIF-8-DOX** exhibited a pH-responsive release capacity derived from **ZIF-8**, and upon irradiation with a laser (980 nm), it further released **DOX**, which indicated the achievement of dual-stimuli-responsive release of **DOX** via pH and NIR laser irradiation. In addition, as shown in Figure 6, **UCNP@ZIF-8-DOX** (concentration: 50 μg mL^–1^) significantly reduced the viability of the tumor cell line (MCF-7) compared with that of the healthy cell line (MCF-10A) upon laser irradiation, which demonstrated that **UCNP@ZIF-8-DOX** enhanced therapeutic efficacy with a decrease in side effects.

**Figure 8:**
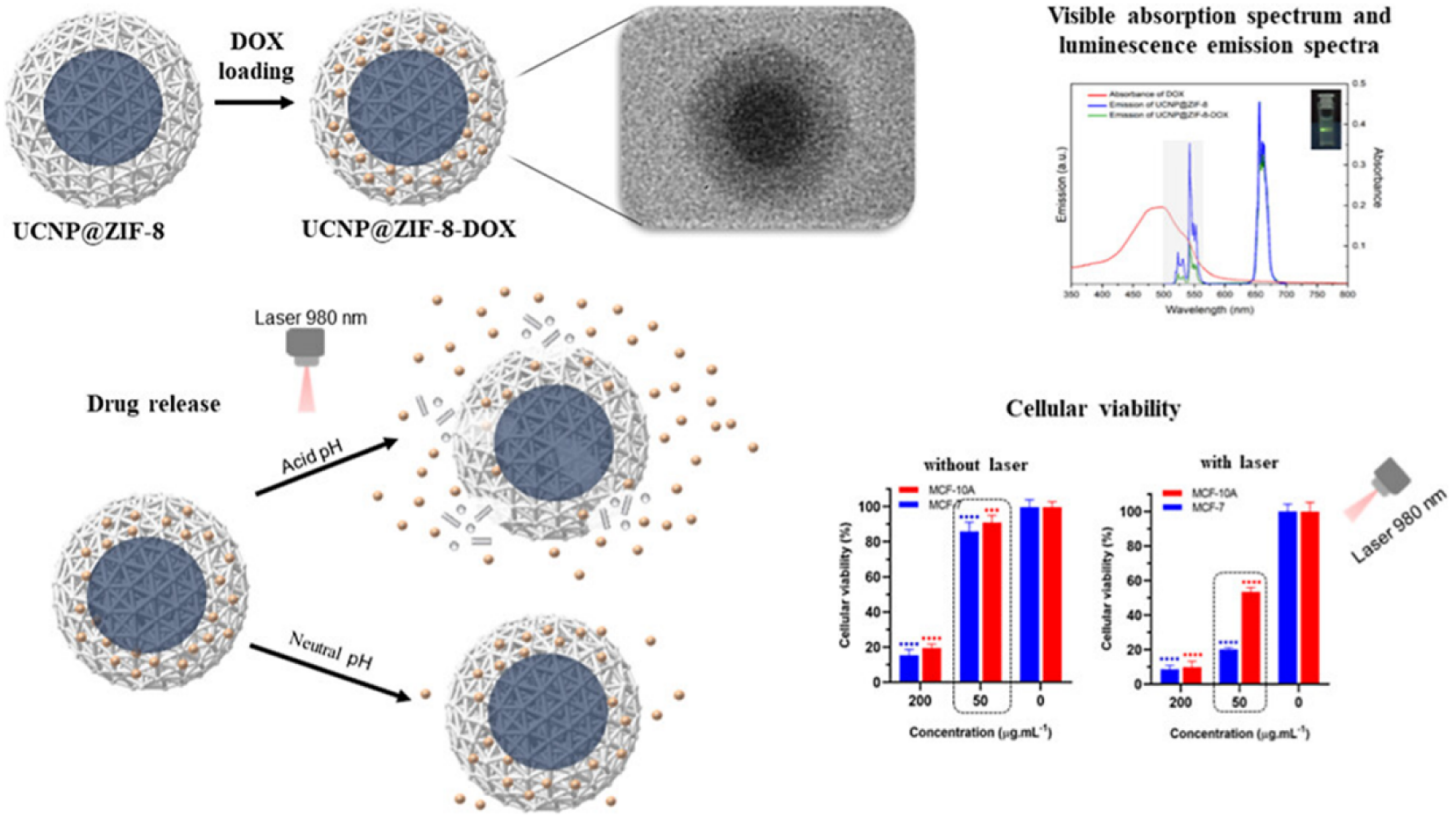
Schematic representation of the preparation of **UCNP@ZiF-8-Dox**. Cell viability of MCF-7 and MCF-10A cells with **UCNP@ZIF-8-DOX** and **UCNP@ZIF-DOX** with a 980 nm laser application. Reproduced from ref. 65. Copyright 2025 American Chemical Society.

Drugs encapsulated into porous frameworks have been developed as nanocarriers for drug-delivery applications. In **DDS**, the nanocarrier’s toxicity leads to side effects. Therefore, for cage-like porous frameworks, using low-toxicity components and employing low-toxicity solvents during synthesis and encapsulation are important. For application in **DDS**, the stability of cage-like porous frameworks in aqueous environments is also important. The poor water resistance of **HOF**s raises concerns about the failure to retain drugs. To enhance therapeutic efficacy, maximization of the drug-loading capacity is desired. In many investigations, drugs have been encapsulated using the bottle-around-ship strategy. In this encapsulation process, a solvent that can occupy the pores is employed, which reduces drug-loading capacity. The pressure-driven strategy was discovered as a solvent-free encapsulation method and demonstrated the encapsulation of ibuprofen as a model with a high loading ratio. However, their applicability to other drugs remains unclear. In addition, although numerous investigations on drug release capacity and side effects have been conducted as performance evaluations of nanocarriers, most are limited to *in vitro* studies. Therefore, the performance of encapsulated drugs under *in vivo* conditions, which are influenced by complex biological factors, has scarcely been investigated.

### 3.3 Encapsulation of Molecular Catalysts

Molecular catalysts, a type of homogeneous catalyst, enable the precise design of their molecular structures, improve their catalytic performance, and therefore realize reactions that are difficult to achieve with heterogeneous catalysts.^12,13^ In contrast, heterogeneous catalysts can be easily separated from the reaction products and reused. To combine the advantages of these homogeneous and heterogeneous catalysts, the heterogenization based on the immobilization of molecular catalysts has been performed.^12,14^

Most metal complex catalysts are subject to degradation, and therefore, encapsulation has been limited to thermal/chemical stable catalysts.^66^ Recently, as shown in Figure 9a, b, and c, Chen et al. encapsulated and isolated the iron complex [(TPA)­Fe^II^-2L]^2+^ (**FeTPA**, 8.0 Å × 10 Å), which decays readily to its dinuclear form under a condition of concentration and temperature and loses its catalytic activity, into the pores of **UiO-66** using the ship-in-a-bottle strategy.^37^ In an elution test using UV–vis spectroscopy, **FeTPA@UiO-66** showed no leakage of **FeTPA**, which indicated that the catalyst was confined into the pores. In addition, the solid-state UV–vis diffuse absorption spectra and cyclic voltammetry curves in Figure 9d and e demonstrate that **FeTPA** in **FeTPA@UiO-66** maintained a mononuclear form after 20 days and that dimerization was successfully suppressed. **FeTPA@UiO-66** exhibited catalytic activity toward C–H activation reactions, but the turnover number was lower than that of the homogeneous catalyst. Therefore, optimization of the pore size is essential to avoiding inhibition of the catalytic reaction.

**Figure 9:**
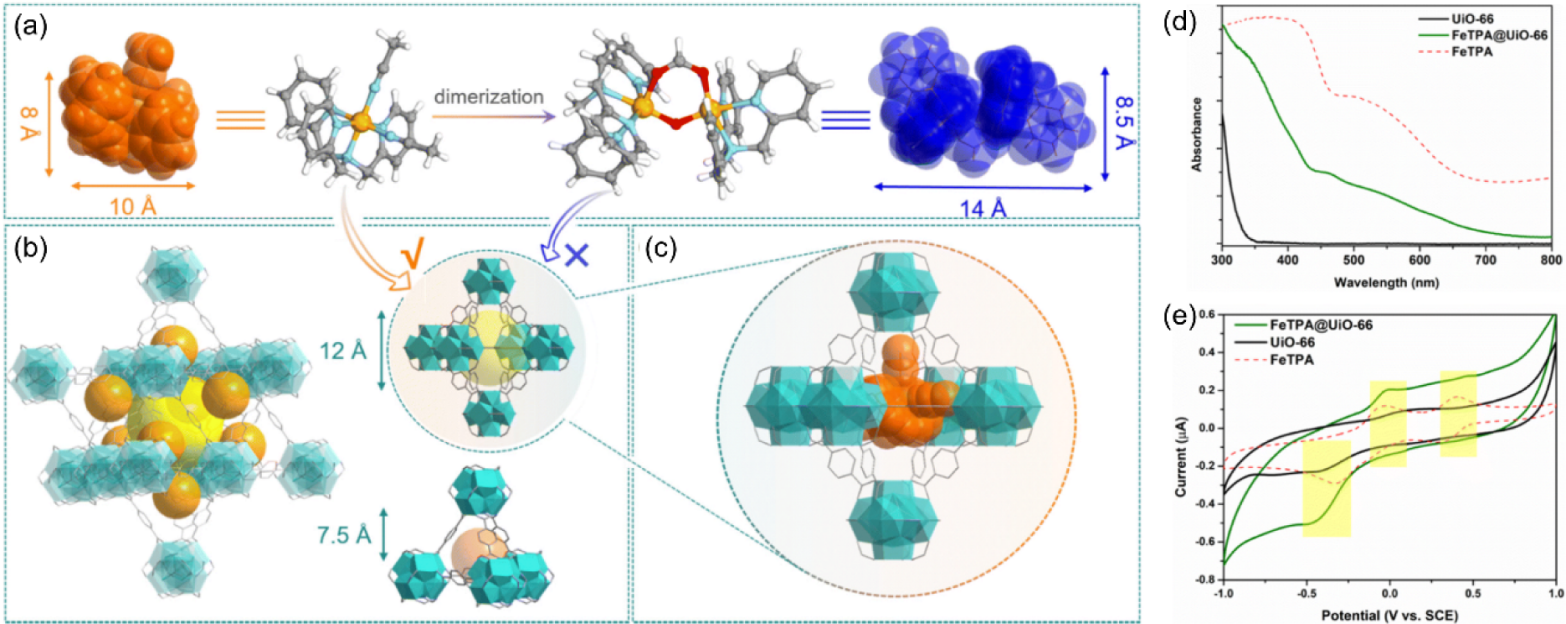
(a) Molecular structures of monomer iron complex [(TPA)­Fe^II^-2L]^2+^ and dimer [Fe^III^_2_(μ-O)­(TPA)_2_(O_2_CH)]^3+^. (b) Illustration of the dimensions and structures of the octahedral and tetrahedral pores in **UiO-66**. (c) Representation of the crystalline structure of **UiO-66** upon encapsulation of mononuclear [(TPA)­Fe^II^-2L]^2+^. (d) Solid-state UV–vis diffuse absorption spectra and (e) **CV** curves in a 100 mM sodium sulfate aqueous solution for **UiO-66**, **FeTPA@UiO-66** (20 days from synthesis), and freshly prepared **FeTPA**. Reproduced with permission from ref. 37. Copyright 2023 Royal Society of Chemistry.

The improvement of the catalytic performance of metal complex catalysts by encapsulation has been reported. As shown in Figures 10a,b, Niu et al. encapsulated the Hoveyda–Grubbs second-generation catalyst (**HG2**, 9.4 Å × 14 Å) and third-generation Grubbs catalyst (**G3**, 11 Å × 14 Å), which are the catalysts for ring-opening metathesis polymerization (**ROMP**), into **UiO-66** and **UiO-67** using the aperture-opening strategy.^38^ The authors synthesized the molecule that connected a luminescent dye to **HG2**, encapsulated it as a model into **UiO-67**, and demonstrated via confocal fluorescence microscopy that the catalyst molecules were encapsulated into the pores. In addition, **G3@UiO-67**, which encapsulated **G3** into **UiO-67**, was applied to the **ROMP** reaction of *cis*-cyclooctene (**COE**) (Figure 10c). As shown in Figure 10d, the cage-like porous structure of **UiO-67** prevented access of the double bond sites in the polymerized **COE** to the catalyst, which suppressed intramolecular and intermolecular secondary metathesis reactions. Consequently, **G3@UiO-67** enabled polymerization with a significantly higher molecular weight and lower dispersity than the homogeneous catalysts. In contrast, the reaction rates and yield were lower than those of the homogeneous **G3**, and the immobilization of molecular catalysts with maintenance of their catalytic performance has been difficult yet.

**Figure 10:**
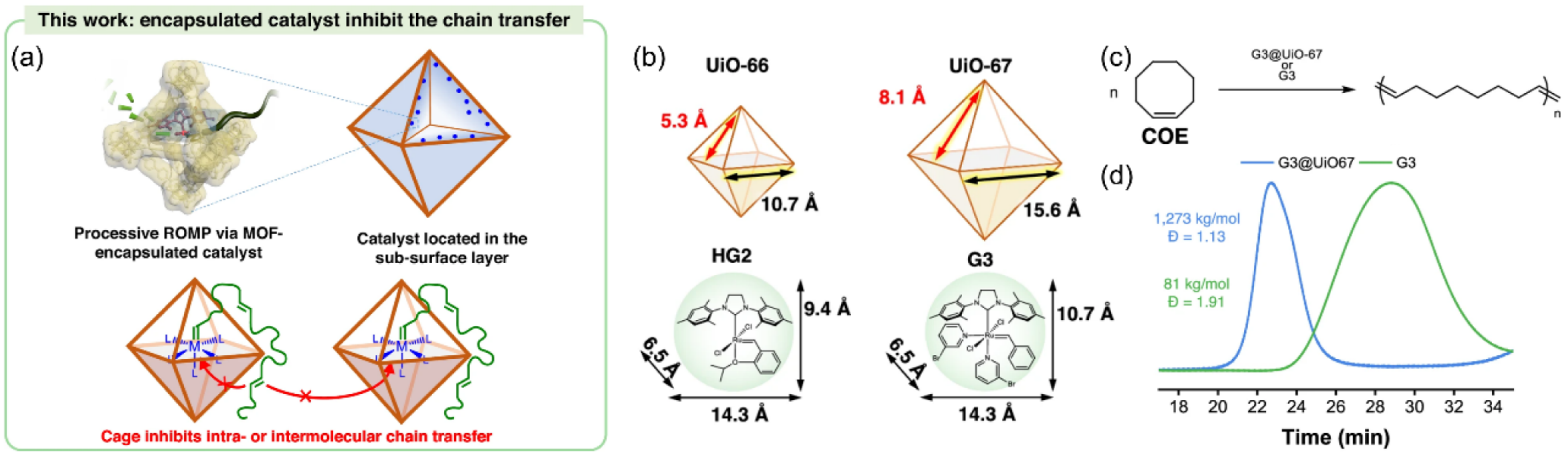
(a) Catalysts are encapsulated in cages of **MOF** crystals. Chain transfer events of the nascent polymers outside of the cages are kinetically inhibited. (b) Size of **HG2** and **G3**, and cavity size of **UiO-66** and **UiO-67**. (c) Equation for ring-opening metathesis polymerization **(ROMP)** of *cis*-cyclooctene (**COE)**. (d) **SEC** traces of the polyoctenamers synthesized by using **G3@UiO-67** and **G3** at comparable monomer conversions. Reproduced from ref. 38. Available under a CC-BY-NC-ND license. Copyright 2025, Z. Zhou, Y. Wang, W. Lo, G. J. Giardino, K. Lalit, M. Goldstein, W. Wang, C. Fields, A. Barney, C. Tsung, U. Mohanty, W. Huang, J. Niu, published by Springer Nature Ltd.

To apply encapsulated catalysts to various reactions, the chemical stability of cage-like porous frameworks is important. Some catalytic reactions require acidic additives. **HOF**s and certain **MOF**s, such as **ZIF**s, collapse in the presence of acids, and therefore, their applicable catalytic reactions can be limited. Therefore, the design strategy of cage-like porous frameworks must take into account their chemical stability under the employed reaction conditions. In the encapsulation of the molecular catalysts, maintenance of the performance (yield and turnover number) of the homogeneous catalysts is desired, but many investigations have failed to achieve this. This is presumably because the narrow window affects the diffusion of the reactants and products. Therefore, the elucidation of the correlation between pore size and catalytic performance is highly desired. In addition, as in **ROMP**, which yields significantly higher molecular weight and lower dispersity, encapsulation may enable new catalytic performance; however, immobilizing molecular catalysts while maintaining their intrinsic catalytic performance remains challenging.^38^ Research on catalytic properties achievable only through encapsulation is still in its early stages, and design guidelines have not yet been established.

## 4 Conclusion and Future Perspectives

This perspective focuses on the encapsulation of functional molecules within cage-like porous frameworks, independent of chemical bonding or chemical interactions between the molecule and the framework, and outlines the characteristics of this encapsulation strategy. In addition, this perspective describes recent advances in the encapsulation of functional molecules and in the development of functional solid materials based on encapsulation (Figure 11).

**Figure 11:**
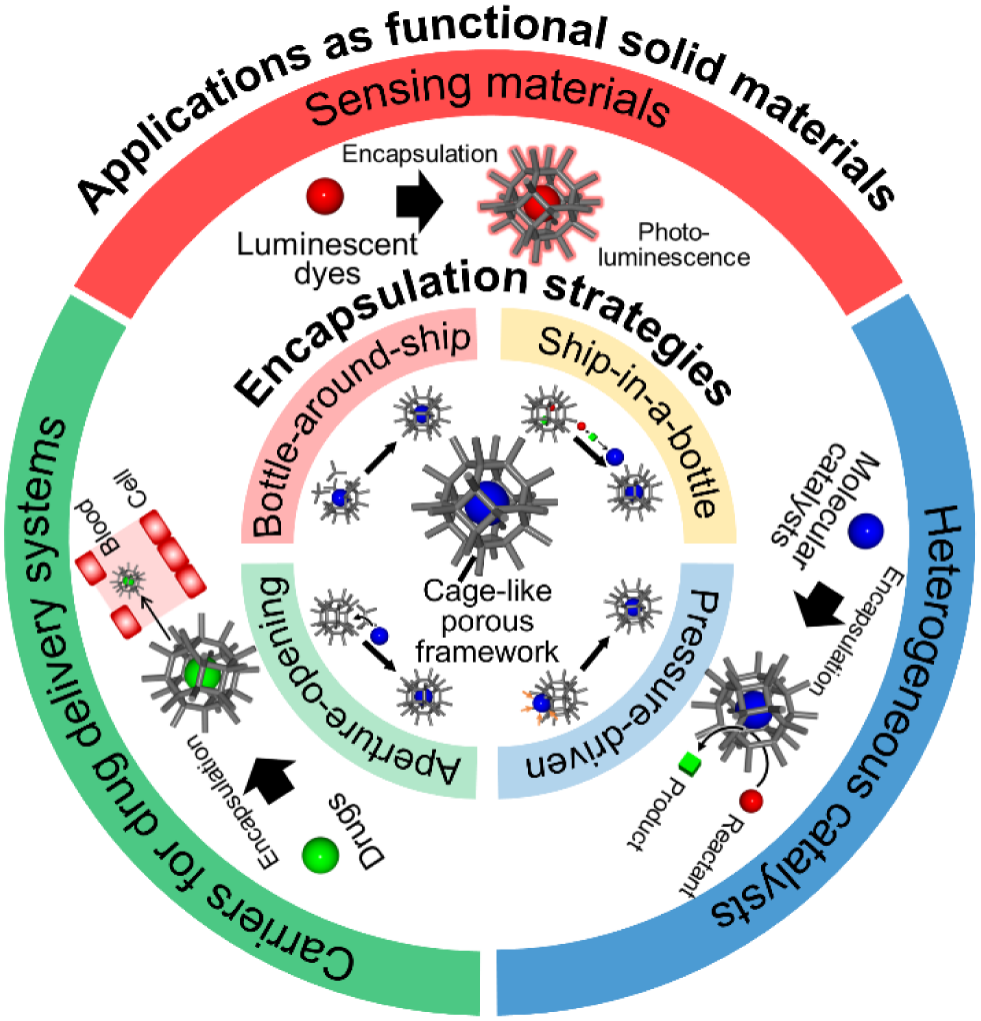
Overview of the encapsulation strategies and their applications.

Section 2 outlines the characteristics of the encapsulation strategies: bottle-around-ship, ship-in-a-bottle, aperture-opening, and pressure-driven strategies. The bottle-around-ship strategy was the primary encapsulation strategy. However, to prevent degradation of functional molecules during encapsulation, cage-like porous frameworks synthesized under mild conditions are preferred. **MOF**s have been used in numerous studies as support materials for the encapsulation of functional molecules; however, **MOF**s that can be synthesized under ambient conditions remain limited. In addition, among the **HOF**s that can be synthesized under relatively mild conditions, cage-like porous structures suitable for encapsulation are rare, and therefore, their design guidelines and control method of their pore sizes have not been established. The ship-in-a-bottle, aperture-opening, and pressure-driven strategies have been developed as postsynthetic encapsulation strategies. In the case of the encapsulation via the ship-in-a-bottle strategy, the narrow windows of cage-like porous frameworks raise concerns about hindering the access of precursor molecules for functional molecules to the inner pores. This concern is expected to be overcome by expanding the window size to exceed the precursor molecules for functional molecules while preventing the leakage of the encapsulated functional molecules. In contrast, aperture-opening and pressure-driven strategies temporarily expand the window during encapsulation via linker dissociation and linker rearrangement under pressure, respectively. This enables the encapsulation of functional molecules, regardless of this concern. In addition, these strategies exhibit different loading ratios and distributions of functional molecules in the crystal, which are considered to affect their performance. Therefore, comparative experiments on the performance of these strategies are highly desired.

Section 3 focuses on luminescent dyes, drugs, and molecular catalysts as functional molecules (Table 2) and summarizes the recent progress in functional solid materials based on their encapsulation.

Luminescent dyes have been encapsulated to avoid quenching based on aggregation in the solid state, and they exhibit luminescence at the same wavelength in the solid state as that in solution. In these systems, the encapsulation extends or shortens the lifetime of the luminescent dyes.^33^ Although this mechanism is considered to involve the conformation of the dye, the specific conformation of the encapsulated dye has not yet been experimentally elucidated. In addition, the pore environment and the adsorption properties of porous frameworks can change the luminescence phenomenon of encapsulated dyes.^42,44^ Although multiple factors likely influence luminescence, detailed elucidation of the mechanisms through dynamic measurements, such as time-resolved transient absorption spectroscopy, has not been achieved.

Drugs have been encapsulated to enhance the drug-loading capacity and to develop nanocarriers that exhibit stimulus-responsive drug release. In **DDS**, the nanocarrier’s toxicity can lead to adverse effects. Therefore, for cage-like porous frameworks, the use of low-toxicity components and solvents during the synthesis and encapsulation is important. To enhance therapeutic efficacy, maximizing the drug-loading capacity is desirable. In many investigations, drugs have been encapsulated using the bottle-around-ship strategy. In this encapsulation process, a solvent that can occupy the pores is employed, which reduces drug-loading capacity. The pressure-driven strategy was identified as a solvent-free encapsulation method and demonstrated the encapsulation of ibuprofen as a model compound at a high loading ratio.^25^ However, its applicability to other drugs remains unclear. In addition, although numerous investigations into drug release capacity and side effects have evaluated nanocarrier performance, most studies have been limited to *in vitro* studies. Therefore, the performance of encapsulated drugs under *in vivo* conditions, which are influenced by complex biological factors, has scarcely been investigated.

Molecular catalysts have been encapsulated to combine the advantages of both homo- and heterogeneous catalysts. Encapsulation that maintains the performance (reaction rate, yield, and turnover number) of the homogeneous catalysts has been difficult yet.^37,38^ This is presumably because the narrow window affects the diffusion of reactants and products. Therefore, elucidation of the correlation between the pore size and catalytic performance is desired. **UiO-66** and **UiO-67** exhibit high chemical stability, which makes them popular cage-like porous frameworks for the encapsulation of catalysts. However, due to their inner pore sizes of less than 20 Å, they are unsuitable for the encapsulation of larger catalysts such as enzymes. In addition, due to their window sizes of less than 9 Å, they are unsuitable for coupling reactions that yield bulky products. To apply encapsulation to a wider variety of catalysts and various catalytic reactions, the use of various **MOF**s with larger inner pore sizes or larger window sizes than those of **UiO-66** and **UiO-67**, is required. In addition, encapsulation may enable new catalytic performance; however, immobilizing molecular catalysts while maintaining their intrinsic catalytic performance remains challenging.^38^ Research on catalytic properties achievable only through encapsulation is still in its early stages, and design guidelines have not yet been established.

In light of the above, the future directions for this research are considered as follows.

First, there is the establishment of design guidelines for pore size and environmental conditions in cage-like porous frameworks suitable for encapsulation. Windows, inner pores, and the pore environment are key factors determining the functional molecules that can be encapsulated. Therefore, the construction of cage-like porous frameworks with systematically varied pore sizes and environments through molecular design of their components and elucidation of the relationship between the pore size and environment with the size of the molecule that can be encapsulated are essential.

Second, there is determination of the optimal loading ratio and pore size to maximize performance. The performance of the encapsulated functional molecules is influenced by their loading ratio and pore size. In particular, the restricted access of reaction substrates and target analytes to the immobilized functional molecules due to the narrow window is a potential disadvantage of encapsulation relative to other immobilization methods, and it is necessary to consider whether the window size is sufficient for the diffusion of reaction substrates and target analytes. This potential disadvantage is expected to be overcome by expanding the window size while preventing leakage of the encapsulated functional molecules. In addition, the optimal value of the loading ratio is expected to differ across the targeted functions. Therefore, elucidating the correlations between the loading ratio and pore size and performance through systematic variation of both parameters is essential for establishing guidelines to maximize performance.

Third, there are the application of these encapsulation strategies to other functional molecules, such as amines, and the realization of novel functions accessible only through encapsulation. The combination of adsorption properties with functional molecules enables the realization of new functions through encapsulation. For example, although amines for CO_2_ capture are easily degraded by O_2_, their encapsulation into porous frameworks with an O_2_ barrier effect endows them with high oxidative stability. To realize these functions, elucidating the correlation between the pore environment and adsorption properties is important, as it enables the development of a method for controlling adsorption properties tailored to cage-like porous frameworks. Systematic research on cage-like porous frameworks and the development of encapsulation strategies will enable the demonstration of the intrinsic or novel functions of functional molecules even within functional solid materials.

Fourth, there is application of amorphous porous frameworks for encapsulation. Toward the practical application, the amorphous porous frameworks such as porous organic polymers (**POP**s) have the potential to decrease their manufacturing costs lower than those of crystalline **MOF**s and **HOF**s. The introduction of functional molecules during the synthesis of **POP**s is expected to induce the formation of a framework around these molecules, thereby achieving their encapsulation. In hollow **POP**s that have spheric hollow in polymer nano spheres, the hollow acts as a cage-like pore to encapsulate functional molecules.^67^ Development of mild preparation conditions for maintenance of their molecular structures and methods to precisely control the window size will broaden the applicability of encapsulation within **POP**s to various functional molecules.

As mentioned above, the immobilization of functional molecules in support materials is an important key to enabling the practical application and industrialization of molecular functions as functional solid materials and systems. Encapsulation enables functional molecules to exhibit their intrinsic molecular functions in the solid state and acquire new functions. For example, when encapsulated molecular catalysts with intrinsic performance are realized, they can be used as fillers in polymer/filler composite membranes, replacing inorganic porous frameworks (such as zeolites and activated carbon) that exhibit low separation selectivity.^68^ In addition, this would enable a membrane reactor capable of both chemical reactions and product purification in a single operation, thereby contributing significantly to energy conservation in the reaction and separation processes. Furthermore, for example, immobilization of dyes in porous frameworks with molecular sieving effects also enables applications in selective sensing (detection) technologies. Further advances in these investigations into the encapsulation of functional molecules could enable innovative detection, separation, and conversion technologies and establish foundational technologies for achieving carbon neutrality and a sustainable society.

## Supplementary Material



## Notes

Table S1: Comparison of loading ratio, stability, and retention of function (drug release) between a typical 3D MOF and a cage-like MOF using caffeine immobilization and release as a model (PDF)
