# Supplementary material for: Encapsulation and Immobilization of Functional Molecules Using Cage-Like Porous Frameworks
Source: ACS Appl Mater Interfaces. 2026 May 5;18(19):27115–27. doi: 10.1021/acsami.6c02300 (PMC13195579; doi:10.1021/acsami.6c02300)
Supplement: Supplementary file 1 [file am6c02300_si_001.pdf]

Supporting Information

Encapsulation and Immobilization of Functional Molecules Using Cage-like Porous Frameworks

Hiroi Sei,[1] Yukako Fujita,[1] Yuta Tanaka,[1] Hitoshi Kasai,[1] and Kouki Oka[*,1,2,3].

[1]Institute of Multidisciplinary Research for Advanced Materials, Tohoku University, 2-1-1 Katahira, Aoba-ku, Sendai, Miyagi 980-8577, Japan.

[2]Center for the Promotion of Interdisciplinary Education and Research, Kyoto University, Yoshida-honmachi, Sakyo-ku, Kyoto 606-8501, Japan.

[3]Carbon Recycling Energy Research Center, Ibaraki University, 4-12-1 Nakanarusawacho, Hitachi, Ibaraki 316-0033, Japan.

Email: oka@tohoku.ac.jp (Kouki Oka)

Table S1. Comparison of loading ratio, stability, and retention of function (drug release) between a typical 3D **MOF** and a cage-like **MOF** using caffeine immobilization and release as a model.

Porous frameworks		Loading ratio of Caffeine (wt %)	Stability (after release experiment in water for 9 days)	Caffeine release duration (day)	Ref
Typical 3D **MOF**s	**MIL-53**	30	Low (Decrease in intensity in the **PXRD** pattern)	9	[1]
Cage-like **MOF**s	**ZIF-8**	28	High (Retention of high intensity in the **PXRD** pattern)	27	[2]

References

(1) Cunha, D.; Ben Yahia, M.; Hall, S.; Miller, S. R.; Chevreau, H.; Elkaïm, E.; Maurin, G.; Horcajada, P.; Serre, C. Rationale of Drug Encapsulation and Release from Biocompatible Porous Metal–Organic Frameworks. *Chem. Mater.* **2013**, *25* (14), 2767-2776.
(2) Liedana, N.; Galve, A.; Rubio, C.; Tellez, C.; Coronas, J. CAF@ZIF-8: one-step encapsulation of caffeine in MOF. *ACS Appl. Mater. Interfaces* **2012**, *4* (9), 5016-5021.
